# Use of Virus Genotypes in Machine Learning Diagnostic Prediction Models for Cervical Cancer in Women With High-Risk Human Papillomavirus Infection

**DOI:** 10.1001/jamanetworkopen.2023.26890

**Published:** 2023-08-02

**Authors:** Ting Xiao, Chunhua Wang, Mei Yang, Jun Yang, Xiaohan Xu, Liang Shen, Zhou Yang, Hui Xing, Chun-Quan Ou

**Affiliations:** 1 Department of Biostatistics, School of Public Health, Southern Medical University, Guangzhou, China; 2 Xiangyang Central Hospital, Affiliated Hospital of Hubei University of Arts and Science, Hubei Province, Xiangyang, China; 3 Department of Epidemiology and Biostatistics, School of Public Health, Guangzhou Medical University, Guangzhou, China; 4 State Key Laboratory of Organ Failure Research, National Clinical Research Center for Kidney Disease, Southern Medical University, Guangzhou, China

## Abstract

**Importance:**

High-risk human papillomavirus (hrHPV) is recognized as an etiologic agent for cervical cancer, and hrHPV DNA testing is recommended as the preferred method of cervical cancer screening in recent World Health Organization guidelines. Cervical cancer prediction models may be useful for screening and monitoring, particularly in low-resource settings with unavailable cytological and colposcopic examination results, but previous studies did not include women infected with hrHPV.

**Objectives:**

To develop and validate a cervical cancer prediction model that includes women positive for hrHPV infection and examine whether the inclusion of HPV genotypes improves the cervical cancer prediction ability.

**Design, Setting, and Participants:**

This diagnostic study included diagnostic data from 314 587 women collected from 136 primary care centers in China between January 15, 2017, and February 28, 2018. The data set was separated geographically into data from 100 primary care centers in 6 districts for model development (training data set) and 36 centers in 3 districts for model validation. A total of 24 391 women identified with positive hrHPV test results in the cervical cancer screening program were included in the study. Data were analyzed from January 1, 2022, to July 14, 2022.

**Main Outcomes and Measures:**

Cervical intraepithelial neoplasia grade 3 or worse (CIN3+) was the primary outcome, and cervical intraepithelial neoplasia grade 2 or worse (CIN2+) was the secondary outcome. The ability of the prediction models to discriminate CIN3+ and CIN2+ was evaluated using the area under the receiver operating characteristic curve (AUROC), sensitivity, specificity, positive likelihood ratio, and negative likelihood ratio. The calibration and clinical utility of the models were assessed using calibration plots and decision curves, respectively.

**Results:**

After excluding women without screening outcomes, the study included 21 720 women (median [IQR] age, 50 [44-55] years). Of 14 553 women in the training data set, 349 (2.4%) received a diagnosis of CIN3+ and 673 (4.6%) of CIN2+. Of 7167 women in the validation set, 167 (2.3%) received a diagnosis of CIN3+ and 228 (3.2%) of CIN2+. Including HPV genotype in the model improved the AUROC by 35.9% for CIN3+ and 41.7% for CIN2+. With HPV genotype, epidemiological factors, and pelvic examination as predictors, the stacking model had an AUROC of 0.87 (95% CI, 0.84-0.90) for predicting CIN3+. The sensitivity was 80.1%, specificity was 83.4%, positive likelihood ratio was 4.83, and negative likelihood ratio was 0.24. The model for predicting CIN2+ had an AUROC of 0.85 (95% CI, 0.82-0.88), with a sensitivity of 80.4%, specificity of 81.0%, positive likelihood ratio of 4.23, and negative likelihood ratio of 0.24. The decision curve analysis indicated that the stacking model provided a superior standardized net benefit when the threshold probability for clinical decision was lower than 23% for CIN3+ and lower than 17% for CIN2+.

**Conclusions and Relevance:**

This diagnostic study found that inclusion of HPV genotypes markedly improved the ability of a stacking model to predict cervical cancer among women who tested positive for hrHPV infection. This prediction model may be an important tool for screening and monitoring cervical cancer, particularly in low-resource settings.

## Key Points

**Question:**

Can human papillomavirus (HPV) screening results and commonly available clinical data be used to develop a high-performance prediction model for cervical cancer among women infected with high-risk HPV?

**Findings:**

In this diagnostic study of 21 720 women with high-risk HPV infection, the developed prediction model had good performance for predicting cervical intraepithelial neoplasia grade 3 or worse and grade 2 or worse, especially when HPV genotype was included in the model.

**Meaning:**

These findings suggest that this prediction model may be an important tool in screening and monitoring cervical cancer, particularly in low-resource settings where high-quality and extensive cytological and colposcopic examinations are unavailable.

## Teaser

This diagnostic study develops and validates a cervical cancer prediction model that includes human papillomavirus (HPV) screening results and commonly available clinical data and assesses whether the inclusion of HPV genotypes improves the cervical cancer prediction ability of the model among women positive for high-risk HPV infection.

## Introduction

Cervical cancer is the fourth most common cancer in women worldwide according to Global Cancer Statistics 2020.^1^ With an estimated 604 000 new cases and 342 000 deaths globally in 2020, cervical cancer poses serious threats to women’s lives and heavy economic burdens on society, especially in developing countries, such as China.^1^ In 2020, China accounted for approximately 18.2% and 17.3% of global new cases of and deaths from cervical cancer, respectively,^2^ highlighting the importance of screening, diagnosis, and management of cervical cancer.

Well-established screening programs for early detection can lead to a clear decrease in the mortality and incidence of cervical cancer. However, the implementation of screening programs in developing countries remains a critical issue.^3,4^ China implemented a national cervical cancer screening program in 2009, but the current coverage rate in China is still less than 30%, with lower rates in rural regions than in urban regions (22.6% vs 30.0%).^5^ In addition, many developing countries, including China, are facing insufficient medical resources and a lack of skilled health care personnel.^6,7^ Therefore, it remains challenging to launch high-quality cervical cancer screening programs covering all women in developing countries. This lack of high-quality screening suggests that the ability to accurately predict cervical cancer based on commonly available clinical information would be of great value in low-resource settings.

Previous studies^8^ have constructed prediction models for cervical cancer based on common clinical information, but the participants were from only 1 or 2 hospitals, which may not be representative of the general population, and the sample sizes were insufficient. In addition, high-risk human papillomavirus (hrHPV) is recognized as an etiologic agent for cervical cancer,^9^ and the different HPV genotypes are associated with different risks of cervical cancer.^10^ It is reasonable to assume that the inclusion of HPV genotypes in the model may improve prediction ability. However, only 2 previous studies have considered HPV genotypes in prediction models. One study considered 3 HPV genotypes (HPV-16, HPV-52, and HPV-35) and observed a model accuracy of 74.4%,^11^ and the other study used HPV genotype classifications of uninfected, low risk, and high risk and observed an area under the receiver operating characteristic curve (AUROC) of 0.73.^12^ A more granular classification of HPV genotypes may further improve the prediction performance.

According to the American Society for Colposcopy and Cervical Pathology (ASCCP) guidelines^13^ and the Chinese Society for Colposcopy and Cervical Pathology guidelines,^14^ women who test negative for hrHPV infection are considered free of the disease, whereas women who test positive for hrHPV infection require subsequent testing during cervical cancer screening. However, approximately 10% to 30% of women who test positive for hrHPV infection do not adhere to the screening procedure for the subsequent cytological or colposcopic examination, and their final cervical lesion status is unknown.^3,15,16^ It is important to predict cervical cancer among this high-risk subpopulation of women infected with hrHPV. But only 1 previous study developed a prediction model for this high-risk subgroup, and the model was not thoroughly evaluated or validated (eg, sensitivity and specificity were not reported).^11^

Most previous studies that developed models to predict cervical cancer used regression models.^8,12,17,18^ Unlike traditional regression models, machine learning approaches do not make assumptions on data distribution and tend to have better predictive performance. The stacking model, also known as a stacked generalization or a super learner, is an ensemble machine learning approach that permits researchers to incorporate several different prediction algorithms, including regression models, and therefore typically performs better than its submodels.^19^ The stacking model has been widely used for the prediction of various diseases and has shown good performance,^20,21,22^ and it may be an appropriate approach to predict cervical cancer.

The aim of the present study was to use a multicenter large-scale data set to develop and validate a stacking machine learning model for predicting cervical cancer among women who tested positive for hrHPV infection by incorporating HPV genotypes and commonly available clinical information. In particular, we examined whether the inclusion of HPV genotypes further improved the prediction ability of the stacking model.

## Methods

### Study Population

From January 15, 2017, to February 28, 2018, a cervical cancer screening program was conducted in 136 primary care centers of Xiangyang City, China. Inclusion criteria for this program were women 30 years or older who had been sexually active for more than 1 year, were not pregnant, and had no previous HPV vaccination or history of hysterectomy or pelvic radiation therapy. This diagnostic study included only women who tested positive for hrHPV infection. All women were interviewed via questionnaires and received a pelvic examination and HPV genotype testing. Women who tested positive for HPV-16/18 infection underwent colposcopy, and women who tested positive for infection with other hrHPV genotypes were referred for a ThinPrep cytological test (Hologic), followed by colposcopy for women who had abnormal ThinPrep cytological test findings. Women with normal ThinPrep cytological test findings were considered free of cervical cancer. Written informed consent was obtained from all participants. The Ethics Committee of Xiangyang Central Hospital reviewed and approved the study, and all procedures followed the ethical standards stipulated by the institution. This study followed the Transparent Reporting of a Multivariable Prediction Model for Individual Prognosis or Diagnosis (TRIPOD) reporting guideline.^23^

### Data Collection

Individual data included participant’s demographic characteristics (age, body mass index, educational level, and insurance type), medical history (history of other cancers and cervical cancer screening history), menstrual status (whether in menopause or age at menopause), sexual behavior factors (gravidity, parity, contraceptive methods, postcoital bleeding, or abnormal leukorrhea), and family history of cancer. The pelvic examination involved the visual inspection of the vulva, internal speculum examination of the vagina and cervix, and bimanual palpation of the adnexa and uterus. We also considered infection of several microorganisms in the vaginal microenvironment, including *Trichomonas vaginalis*, *Candida*, *Gardnerella vaginalis*, and clue cells. Additionally, the status of the vagina as assessed by the number of white blood cells, vaginal bacteria, and miscellaneous bacteria in vaginal secretions was classified into 4 categories, with categories I and II considered normal and III and IV, abnormal.^24^ The HPV genotypes were detected using the Cobas 4800 HPV test (Roche Diagnostics) and included 7 categories: HPV-16, HPV-18, other hrHPV genotypes, HPV-16 plus HPV-18, HPV-16 plus other hrHPV genotypes, HPV-18 plus other hrHPV genotypes, and HPV-16 plus HPV-18 plus other hrHPV genotypes.

Cervical intraepithelial neoplasia 3 or worse (CIN3+) was considered the primary outcome because there is widespread agreement that detecting and treating CIN3+, an important premalignant condition of the cervix, can prevent the progression to invasive cervical cancer.^25^ Cervical intraepithelial neoplasia 2 or worse (CIN2+) was the secondary outcome because it is a treatment threshold and a commonly used outcome in published studies.^12,17,18^

### Training and External Validation Set Splitting by Geography

Geographic validation as a form of external validation was used to assess the prediction performance of the model, with 100 primary care centers in China (6 districts: Laohekou, Nanzhang, Xiangcheng, Xiangzhou, Yicheng, and Zaoyang) used for model development and the remaining 36 primary care centers (3 districts: Baokang, Gucheng, and Fancheng) used for model validation.

### Statistical Analysis

Women without cervical cancer screening outcomes were excluded from the analysis. We also excluded potential predictors that had more than 50% missing values. In both the training and validation data sets, all of the remaining predictors had less than 5% missing values, and the missing data were not imputed.

The continuous predictors (age, gravidity, and parity) were normalized using the equation Z = (*X*–*X_a_*)/(*X_b_*–*X_a_*), where *X*_a_ is the minimum value and *X*_b_ is the maximum value, to speed the computation of machine learning models during the learning phase. The least absolute shrinkage and selection operator (LASSO) method was used to select predictors that had a potential association with the outcomes.

The stacking machine learning model was constructed to predict cervical cancer among women who tested positive for hrHPV infection, with age and other factors selected by LASSO included as predictors. The stacking model was composed of 2 layers. A traditional statistical method; a logistic regression model; and 4 extensively used machine learning algorithms, including random forest, gradient boosting machine, naive Bayes, and neural network, were selected for the first layer, with the logistic regression model considered the second layer model (eFigure 1 in Supplement 1). To examine whether the inclusion of HPV genotypes could improve predictive ability, models with or without HPV genotypes were separately fitted. Each prediction algorithm was built with 3 groups of predictors (only epidemiological factors and pelvic examination results; only HPV genotypes; and all predictors).

We assessed the performance of the stacking model and its submodel in terms of discrimination, calibration, and clinical utility. The AUROC was used to reflect the discrimination of prediction models. Sensitivity, specificity, positive likelihood ratio, and negative likelihood ratio were calculated using the maximum Youden index criterion. The calibration plot was then used to examine the agreement between model predictions and observed outcomes. Finally, we evaluated the clinical utility of the prediction models using a decision curve analysis.^26^ Based on the assumption that referral for colposcopy brought benefits to women with CIN3+ (or CIN2+) and brought harm to women without CIN3+ (or CIN2+), the decision curve analysis quantified the standardized net benefit (SNB) among hrHPV-positive women based on the prediction model as compared with the default strategy that none receives the intervention (ie, referral to colposcopy). Using the true-positive rate (*TPR*), false-positive rate (*FPR*), clinical decision threshold probability (*R*), and disease prevalence (*P*), the SNB can be calculated as SNB = *TPR* − (*FPR* × [*R*/{1 − *R*}] × [({1 − *P*}/*P*]). Individuals would accept the intervention when their predictive probabilities were greater than *R,* which is affected by clinicians’ and patients’ preferences.

In the cervical cancer screening program, women who were positive for other hrHPV genotypes but had normal cytological examination results were considered free of cervical cancer.^14,27^ A sensitivity analysis was conducted to assess whether potential misclassification moderated the results. Based on the mean incidence rates of CIN3+ and CIN2+ reported by a meta-analysis,^28^ we randomly sampled 0.3% and 0.8%, respectively, of women positive for other hrHPV genotypes but who had normal cytological examination results in the validation set. The outcomes of these subsets were categorized as CIN3+ (or CIN2+). Then the prediction performance of the model was reevaluated.

Statistical analyses were performed from January 1, 2022, to July 14, 2022. A 2-sided value of *P* < .05 or the 95% CI of the AUROC excluding 0.5 was considered statistically significant. All statistical analyses were conducted with R, version 4.1.1 (R Foundation for Statistical Computing), and the stacking model was implemented using the free and open-source package h2o in R. The R code and a user guide are provided in the eAppendix in Supplement 1.

## Results

### Selection of Participants

**Figure 1.**
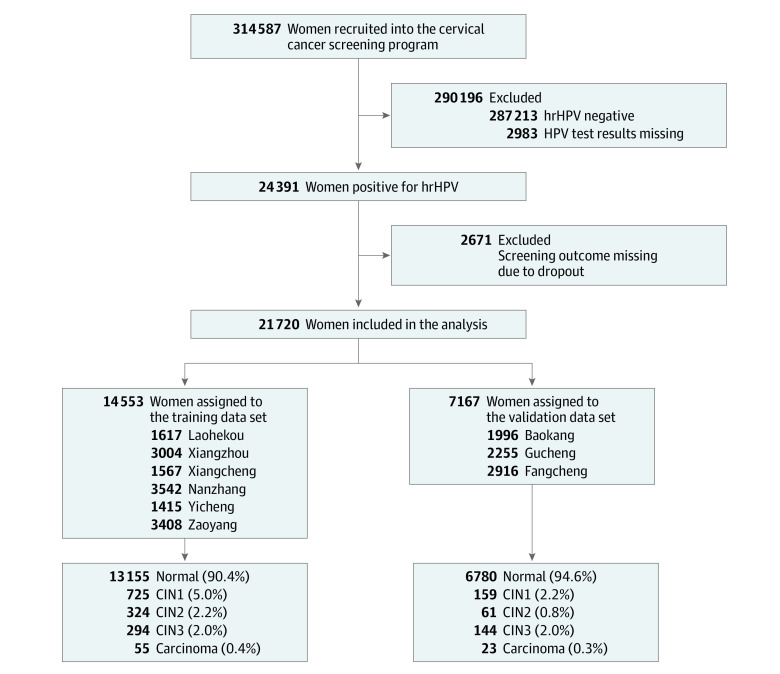
Study Flowchart CIN1 indicates cervical intraepithelial neoplasia grade 1; CIN2, cervical intraepithelial neoplasia grade 2; CIN3, cervical intraepithelial neoplasia grade 3; and hrHPV, high-risk human papillomavirus.

Figure 1 is a flowchart of the selection of women in the study. A total of 314 587 women participated in the cervical cancer screening, and 24 391 (7.8%) were infected with hrHPV. We excluded 2671 (11.0%) women who were hrHPV positive without screening outcomes due to dropout, and the remaining 21 720 women (89.0%; median [IQR] age, 50 [44-55] years) were included in our analysis. There were 14 553 women in the training data set, of whom 349 (2.4%) received a diagnosis of CIN3+, and 673 (4.6%) received a diagnosis of CIN2+. The validation data set comprised 7167 women, with 167 (2.3%) identified as having CIN3+ and 228 (3.2%) identified as having CIN2+. The median (IQR) ages of women were 51 (44-56) years in the training data set and 49 (43-55) years in the validation data set. In the training data set, other hrHPV genotypes accounted for the highest percentage (77.2%) of genotypes, followed by HPV-16 (11.8%), HPV-16 plus other hrHPV genotypes (5.2%), HPV-18 (3.5%), HPV-18 plus other hrHPV genotypes (1.6%), HPV-16 plus HPV-18 (0.3%), and HPV-16 plus HPV-18 plus other hrHPV genotypes (0.3%). A similar distribution of HPV genotypes was found in the validation data set (eTable 1 in Supplement 1).

### Predictors Selected by LASSO

The Table presents the summary statistics of predictors selected by LASSO for CIN3+ status in the training and validation data sets. Statistically significant differences in the numbers and percentages of women between the less severe than CIN3+ (<CIN3) group of 14 204 women (including normal, CIN1, and CIN2) and the CIN3+ group of 349 women were found in the training set for abnormal cervix detected in pelvic examination (5091 [36.1%] for <CIN3 vs 209 [60.4%] for CIN3+; *P* < .001), vaginal status (eg, category III-IV, 4445 [32.8%] for <CIN3 vs 141 [42.2%] for CIN3+; *P* < .001), and HPV genotypes (eg, HPV-16, 1511 [10.6%] for <CIN3 vs 213 [61.0%] for CIN3+; *P* < .001), as well as in the validation data set. Gravidity (median [IQR], 2 [2-3] for <CIN3 vs 3 [2-4] for CIN3+; *P* = .004) and family history of cancer (eg, No. [%] for yes, 267 [1.9%] for <CIN3 vs 14 [4.0%] for CIN3+; *P* = .008) showed statistically significant differences in the training data set, whereas postcoital bleeding (eg, No. [%] for yes, 97 [1.4%] for <CIN3 vs 7 [4.2%] CIN3+; *P* = .008) showed statistically significant differences only in the validation data set. Similar differences were observed for the predictors of CIN2+ (eTable 2 in Supplement 1).

**Table.** Predictors Selected by LASSO for CIN3+

Predictor	Participants in training the data set, No. (%)				Participants in the validation data set, No. (%)			
	Overall	<CIN3^a^	CIN3+^a^	*P* value	Overall	<CIN3^a^	CIN3+^a^	*P* value
No.	14 553	14 204	349	NA	7167	7000	167	NA
Age, median (IQR), y	51 (44-56)	51(44-56)	49 (44-55)	.11	49 (43-55)	49 (43-55)	48 (43-54)	.26
Gravidity, median (IQR)	2 (2-3)	2 (2-3)	3 (2-4)	.004	2 (2-3)	2 (2-3)	2 (2-3)	.73
**Family history of cancer**								
No	14 272 (98.1)	13 937 (98.1)	335 (96.0)	.008	6997 (97.6)	6837 (97.7)	160 (95.8)	.19
Yes	281 (1.9)	267 (1.9)	14 (4.0)		170 (2.4)	163 (2.3)	7 (4.2)	
**Postcoital bleeding**								
No	14 448 (99.3)	14 105 (99.3)	343 (98.3)	.06	7063 (98.5)	6903 (98.6)	160 (95.8)	.008
Yes	105 (0.7)	99 (0.7)	6 (1.7)		104 (1.5)	97 (1.4)	7 (4.2)	
**Pelvic examination: cervix**								
Normal	9135 (63.3)	8998 (63.9)	137 (39.6)	<.001	3938 (55.2)	3885 (55.7)	53 (31.7)	<.001
Abnormal	5300 (36.7)	5091 (36.1)	209 (60.4)		3200 (44.8)	3086 (44.3)	114 (68.3)	
**Hysteromyoma**								
No	14 470 (99.4)	14 121 (99.4)	349 (100)	.28	7080 (98.8)	6917 (98.8)	163 (97.6)	.29
Yes	83 (0.6)	83 (0.6)	0		87 (1.2)	83 (1.2)	4 (2.4)	
**Vaginal status** ^b^								
I-II	9309 (67.0)	9116 (67.2)	193 (57.8)	<.001	4697 (67.4)	4606 (67.7)	91 (54.8)	.001
III-IV	4586 (33.0)	4445 (32.8)	141 (42.2)		2277 (32.6)	2202 (32.3)	75 (45.2)	
***Candida*** ^b^								
No	13 420 (95.7)	13 102 (95.7)	318 (94.4)	.28	6650 (95.2)	6490 (95.2)	160 (96.4)	.59
Yes	603 (4.3)	584 (4.3)	19 (5.6)		336 (4.8)	330 (4.8)	6 (3.6)	
***Gardnerella vaginalis*** ^b^								
No	13 965 (99.6)	13 628 (99.6)	337 (100)	.44	6956 (99.6)	6791 (99.6)	165 (99.4)	>.99
Yes	58 (0.4)	58 (0.4)	0		30 (0.4)	29 (0.4)	1 (0.6)	
**Clue cells** ^b^								
No	13 827 (98.6)	13 499 (98.6)	328 (97.3)	.08	6833 (97.8)	6671 (97.8)	162 (97.6)	>.99
Yes	196 (1.4)	187 (1.4)	9 (2.7)		153 (2.2)	149 (2.2)	4 (2.4)	
**HPV genotypes**								
16 Only	1724 (11.8)	1511 (10.6)	213 (61.0)	<.001	830 (11.6)	733 (10.5)	97 (58.1)	<.001
16 + 18	43 (0.3)	41 (0.3)	2 (0.6)		12 (0.2)	10 (0.1)	2 (1.2)	
16 + 18 + Other hrHPV genotypes	48 (0.3)	46 (0.3)	2 (0.6)		11 (0.2)	10 (0.1)	1 (0.6)	
16 + Other hrHPV genotypes	761 (5.2)	704 (5.0)	57 (16.3)		392 (5.5)	360 (5.1)	32 (19.2)	
18 Only	509 (3.5)	499 (3.5)	10 (2.9)		224 (3.1)	212 (3.0)	12 (7.2)	
18 + Other hrHPV genotypes	236 (1.6)	234 (1.6)	2 (0.6)		102 (1.4)	100 (1.4)	2 (1.2)	
Other hrHPV genotypes	11 232 (77.2)	11 169 (78.6)	63 (18.1)		5596 (78.1)	5575 (79.6)	21 (12.6)	

Abbreviations: CIN3, cervical intraepithelial neoplasia grade 3; hrHPV, high-risk human papillomavirus; LASSO, least absolute shrinkage and selection operator; NA, not applicable.

^a^ Better than CIN3 (<CIN3), includes normal, CIN1, and CIN2; CIN3+ includes CIN3 and carcinoma.

^b^ Predictors with missing values.

### Discrimination of Prediction Models

**Figure 2.**
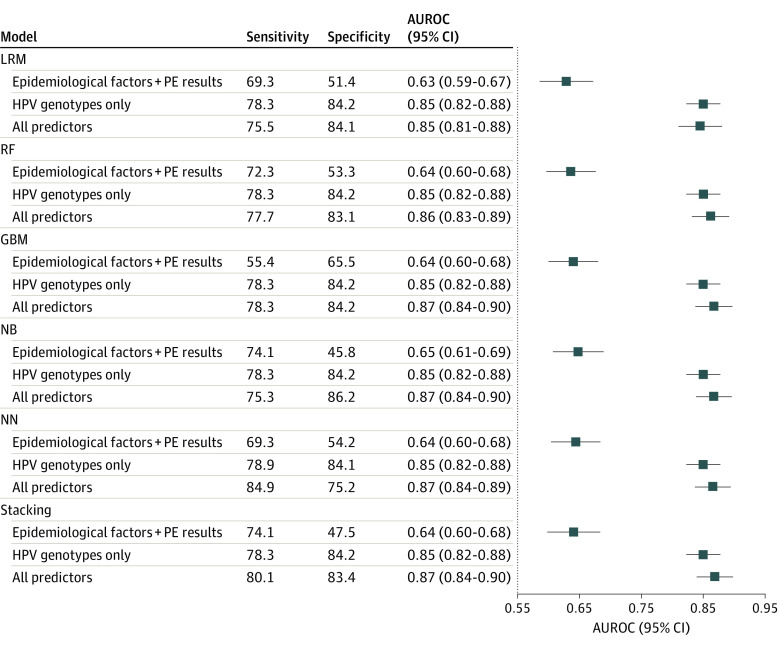
Area Under the Receiver Operating Characteristic Curve (AUROC), Sensitivity, and Specificity of Prediction Models for Predicting Cervical Intraepithelial Neoplasia Grade 3 or Worse in the Validation Data Set GBM indicates gradient boosting machine; HPV, human papillomavirus; LRM, logistic regression model; NB, naive Bayes; NN, neural network; PE, pelvic examination; and RF, random forest.

Figure 2 illustrates the AUROC, sensitivity, and specificity of 6 prediction models with different factors for predicting CIN3+ among hrHPV-positive women. All performances were evaluated using the validation data set. Consistently for all models, the AUROC values of models that included only epidemiological factors and pelvic examination results were approximately 0.64, which was improved by 35.9% ([0.87 − 0.64]/0.64) with the additional inclusion of HPV genotypes in the models. On the other hand, with the inclusion of HPV genotypes in the stacking model, further adding epidemiological factors and pelvic examination results improved the prediction ability (*P* = .02; DeLong test), but the improvement was not marked, with the AUROC changing from 0.85 (95% CI, 0.82-0.88) to 0.87 (95% CI, 0.84-0.90). The stacking machine learning model with all predictors included had an AUROC of 0.87 (95% CI, 0.84-0.90), with sensitivity of 80.1%, specificity of 83.4%, positive likelihood ratio of 4.83, and negative likelihood ratio of 0.24 (Figure 2 and eTable 3 in Supplement 1). When predicting CIN2+, including HPV genotype in the model improved the AUROC by 41.7% ([0.85 − 0.60]/0.60). The stacking model including all predictors also performed best, with an AUROC of 0.85 (95% CI, 0.82-0.88), sensitivity of 80.4%, specificity of 81.0%, positive likelihood ratio of 4.23, and negative likelihood ratio of 0.24 (eFigure 2 and eTable 3 in Supplement 1).

### Calibration of Prediction Models

The naive Bayes model overestimated the risk of CIN3+, whereas the other 5 models were well calibrated. When predicting CIN2+, all models slightly overpredicted the risk of CIN2+ (eFigure 3 in Supplement 1).

### Clinical Utility of Prediction Models

**Figure 3.**
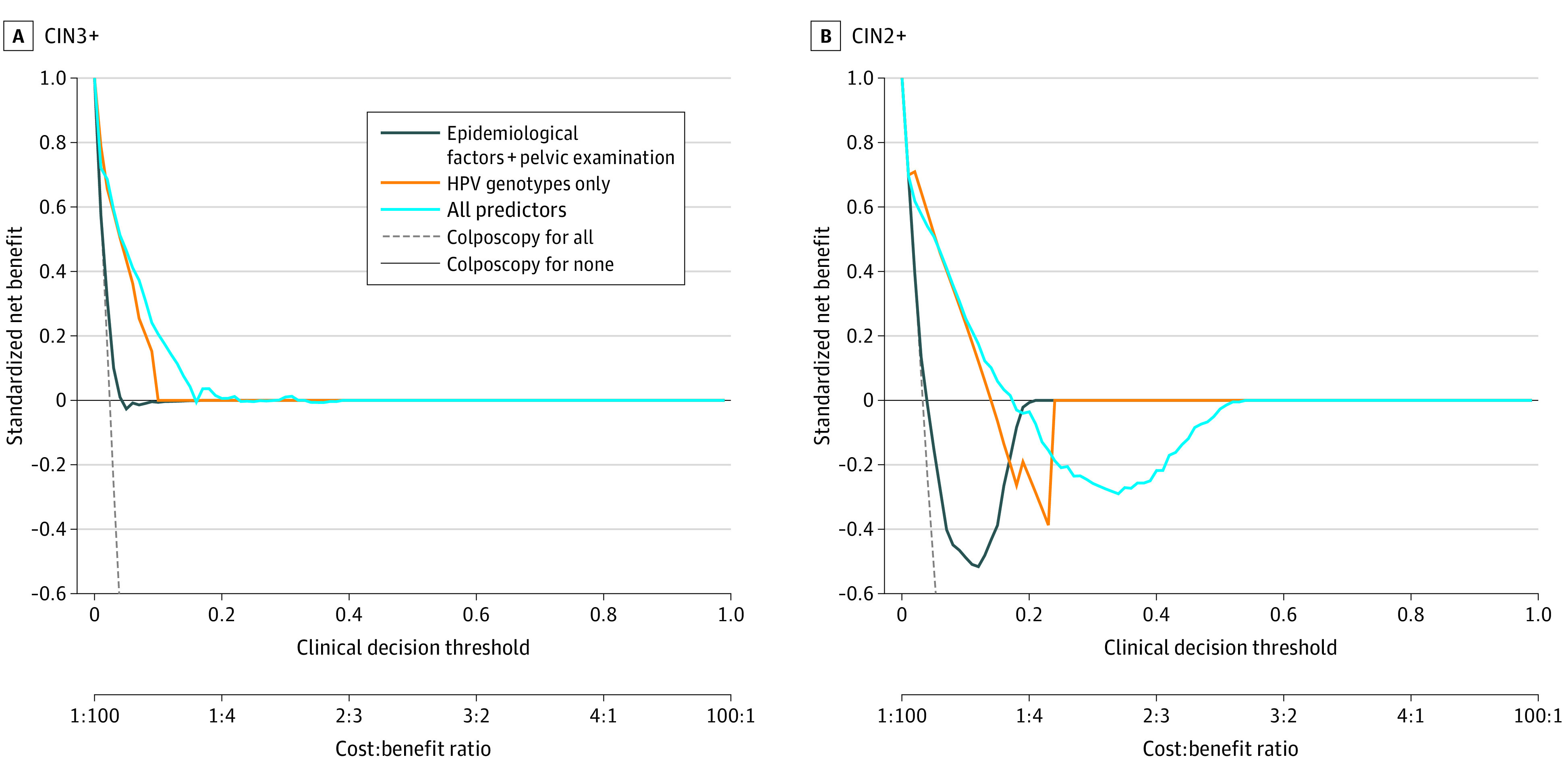
Decision Curves of the Stacking Models With Different Factors Used for Predicting Cervical Intraepithelial Neoplasia Grade 3 or Worse (CIN3+) and CIN2+ Among Women Positive for High-Risk Human Papillomavirus (HPV) Infection The vertical dashed line represents the standardized net benefit for the strategy in which all women underwent colposcopy (colposcopy for all); solid black horizontal line (at 0 on the y-axis), the standardized net benefit for the strategy in which none of the women underwent colposcopy (colposcopy for none).

Figure 3 displays population SNBs of the stacking models with different predictors by comparison with the strategy in which none of the women underwent colposcopy (colposcopy for none). All prediction models had similar SNBs with the strategy in which all women underwent colposcopy (colposcopy for all) when the threshold probability was below about 4%. Additional inclusion of HPV genotypes in the stacking model achieved the greatest clinical value when clinical decision thresholds were lower than 23% for CIN3+ and lower than 17% for CIN2+.

### Sensitivity Analysis

In the sensitivity analysis, a proportion of women who tested positive for other hrHPV genotypes but had normal cytological examination results were categorized as belonging to the CIN3+ (or CIN2+) group (the validation set). We found that the specificity of the 6 models did not change (range, 74.2%-86.2%), while the AUROC (range, 0.82-0.84) and sensitivity (range, 64.3%-80.4%) slightly decreased compared with the main analysis (eTable 4 in Supplement 1).

## Discussion

To our knowledge, this diagnostic study is the first to build a cervical cancer diagnostic prediction model for women with hrHPV infection. A stacking machine learning model that included epidemiological factors, pelvic examination results, and HPV genotypes predicted CIN3+ and CIN2+ well, outperforming previous models that included only common clinical information.^29,30^ The inclusion of HPV genotypes played an important role in improving prediction ability. The diagnostic prediction model developed in this study performed well in identifying women at high risk of CIN3+ or CIN2+. This ability may strengthen patients’ risk awareness and help physicians target women at high risk of cervical cancer to suggest further immediate screening (cytological or colposcopic examination), which may increase screening compliance. In low-resource settings, especially in areas where cytological and colposcopic examinations are unavailable, the developed prediction model using HPV genotyping may be a practical tool for cervical cancer screening.

The epidemiological factors (demographic characteristics, medical history, menstrual status, sexual behavior factors, and family history of cancer) and pelvic examination results considered in this study can be easily obtained at low cost in clinical settings. Therefore, the model can be used in low-resource settings when data on HPV genotypes are available. In addition to epidemiological factors, some biomarkers, such as vascular endothelial growth factor^31^ and HPV-E6/E7 mRNA,^32,33^ have been considered in some cervical cancer prediction models. Although adding biomarkers as predictors may improve the prediction performance of models,^34^ it is difficult to popularize biomarker acquisition technology in primary care settings due to shortages of technicians and equipment and the relatively high costs. This complex and expensive technology has limited practical applications in undeveloped regions or for large-scale population screening programs.

The present study highlighted the importance of HPV genotype in developing cervical cancer. The HPV genotype was a dominant predictor in our model. Compared with the stacking model that included only epidemiological factors and pelvic examination results, the AUROC of the model incorporating HPV genotypes increased by 35.9% for CIN3+ and 41.7% for CIN2+. We found that with the inclusion of HPV genotypes in the model, further adding epidemiological factors and pelvic examination results only slightly improved the prediction ability. Some previous studies also considered HPV infection data when constructing prediction models.^8^ For instance, Rothberg et al^17^ used HPV infection or not as a predictor, van der Waal et al^12^ classified HPV infection into 2 categories of risks (low vs high), and Lee et al^18^ used hrHPV DNA load as an indicator. In our study, we used a more granular classification of HPV genotypes that included 7 categories: HPV-16, HPV-18, other hrHPV genotypes, and 4 coinfections, which are the most common HPV test results in China. Technology for HPV self-sampling is currently being promoted^35^ and is expected to expand the application of this prediction model based on HPV genotype in the future. Women could collect samples themselves and send the samples to specialized laboratories for testing to ensure accuracy.

With the advantage of high-throughput assays and easy sampling, HPV-DNA testing is recommended as the preferred method of cervical cancer screening in recent World Health Organization guidelines.^36^ The prediction model in our study can be used as a triage tool after HPV testing to identify groups with a particularly high risk of cervical cancer. The sensitivity of the model was 80.1% for CIN3+ and 80.4% for CIN2+. There are 2 screening strategies for HPV combined with cytology that are commonly used in China^37^: one approach is HPV with reflex to cytology, that is, women who test positive for HPV infection and have abnormal cytology are recommended for immediate colposcopy, whereas women with a positive HPV test but normal cytology are retested later. The other approach involves HPV genotypes and reflex cytology, in which women with an HPV-16/18 positive test result or who test positive for other hrHPV genotypes but display abnormal cytology results undergo colposcopy, whereas women positive for other hrHPV genotypes but with normal cytology are retested at intervals. A study by Wang et al^37^ compared these 2 screening strategies in China and found that for CIN3+ and CIN2+, the sensitivities of the first strategy were 60.1% and 55.7%, respectively, whereas the sensitivities of the second strategy were 83.2% and 81.7%, respectively. A study in the United States found that the first strategy had a sensitivity of 51.9% and 47.5% for CIN3+ and CIN2+, respectively, whereas the second strategy had a sensitivity of 72.0% and 63.6% for CIN3+ and CIN2+, respectively.^38^ However, the specificity of the prediction model in the present study was lower than those of the 2 screening strategies used by both of these studies. The specificities of our prediction model for CIN3+ and CIN2+ were 83.4% and 81.0%, respectively. Wang et al^37^ reported that the first strategy had a specificity of 98.2% and 99.0%, and the second strategy had a specificity of 98.0% and 98.8% for CIN3+ and CIN2+, respectively. In the study conducted in the United States, the specificities for CIN3+ and CIN2+ were 85.7% and 91.8% for the first strategy and 85.2% and 91.3% for the second strategy for CIN3+ and CIN2+, respectively. The higher specificity observed in those studies may be partly because sequential tests improve the specificity of the diagnosis. The aforementioned strategies calculated the specificity among all participants, including many healthy women, whereas the prediction model used in the present study focused on hrHPV-positive women had difficulties identifying true-negative participants. The prediction model in the present study did not need information on cytology, but the sensitivity of our prediction model was much higher than that of the first strategy and was similar to that of the second strategy.

The stacking model achieved superior net benefit when the clinical decision threshold was below 23% and 17% for CIN3+ and CIN2+, respectively. The ASCCP recommends a 4% threshold, that is, women with a predicted risk of CIN3+ higher than 4% are recommended for colposcopy.^39^ Using the decision curves generated in the present study, when the threshold was 4% or lower, the SNB was approximately 50% for all models. Our model had significantly higher SNB than the strategy of colposcopy for all when the threshold was above 4%. Physicians may have different threshold preferences because of different perspectives on the relative harms of missing cervical cancer vs avoiding unnecessary colposcopy. However, within reasonable clinical thresholds, the SNB for the stacking model was superior to the strategies of colposcopy for all or colposcopy for none and may be used in clinical practice.

### Limitations

This study has limitations. First, some of the predictors were obtained through a self-reported questionnaire, which may lead to reporting bias and recall bias. Second, some factors, such as smoking and oral contraceptive use, which have been documented to potentially influence cervical cancer,^40^ were not collected in the present study. Third, the screening program included only women 30 years of age or older, and the risk factors for cervical cancer may differ in women younger than 30 years. Therefore, the performance of this prediction model in younger women needs to be validated in future studies.

## Conclusions

This diagnostic study developed and validated a diagnostic prediction model for cervical cancer among women who test positive for hrHPV infection. Including HPV genotypes in the model markedly improved the prediction ability, suggesting that this prediction model may be an important auxiliary tool in screening for and early diagnosis of cervical cancer in low-resource settings when cytological and colposcopic examination results are unavailable.

## Notes

Supplement 1. **eTable 1.** Candidate Predictors and Screening Outcomes in the Training and Validation Data Set **eTable 2.** Predictors Selected by LASSO for CIN2+ **eTable 3.** Positive Likelihood Ratio and Negative Likelihood Ratio for All Models **eTable 4.** Results of Sensitivity Analysis **eFigure 1.** The Construct-Diagram of the Stacking Model **eFigure 2.** Area Under the Receiver Operating Characteristic Curve (AUROC), Sensitivity, and Specificity of Prediction Models for Predicting Cervical Intraepithelial Neoplasia Grade 2 or Worse in the Validation Data set **eFigure 3.** Calibration Plots of the Prediction Models Incorporating Epidemiological Factors, Pelvic Examination Results, and Human Papillomavirus (HPV) Genotypes for Predicting the Cervical Intraepithelial Neoplasia Grade 3 or Worse (CIN3+) and CIN2+ Among Women Positive for High-Risk HPV Infection **eAppendix.** R Code and User Guide

Supplement 2.
Data Sharing Statement
