# Supplementary material for: Use of Virus Genotypes in Machine Learning Diagnostic Prediction Models for Cervical Cancer in Women With High-Risk Human Papillomavirus Infection
Source: JAMA Netw Open. 2023 Aug 2;6(8):e2326890. doi: 10.1001/jamanetworkopen.2023.26890 (PMC10398410; doi:10.1001/jamanetworkopen.2023.26890)
Supplement: Supplement 1. — eTable 1. Candidate Predictors and Screening Outcomes in the Training and Validation Data Set eTable 2. Predictors Selected by LASSO for CIN2+ eTable 3. Positive Likelihood Ratio and Negative Likelihood Ratio for All Models eTable 4. Results of Sensitivity Analysis eFigure 1. The Construct-Diagram of the Stacking Model eFigure 2. Area Under the Receiver Operating Characteristic Curve (AUROC), Sensitivity, and Specificity of Prediction Models for Predicting Cervical Intraepithelial Neoplasia Grade 2 or Worse in the Validation Data set eFigure 3. Calibration Plots of the Prediction Models Incorporating Epidemiological Factors, Pelvic Examination Results, and Human Papillomavirus (HPV) Genotypes for Predicting the Cervical Intraepithelial Neoplasia Grade 3 or Worse (CIN3+) and CIN2+ Among Women Positive for High-Risk HPV Infection eAppendix. R Code and User Guide [file jamanetwopen-e2326890-s001.pdf]

Supplementary Online Content

Xiao T, Wang C, Yang M, et al. Use of virus genotypes in machine learning diagnostic prediction models for cervical cancer in women with high-risk human papillomavirus infection. *JAMA Netw Open*. 2023;6(8):e2326890. doi:10.1001/jamanetworkopen.2023.26890

eTable 1. Candidate Predictors and Screening Outcomes in the Training and Validation Data Set
eTable 2. Predictors Selected by LASSO for CIN2+
eTable 3. Positive Likelihood Ratio and Negative Likelihood Ratio for All Models
eTable 4. Results of Sensitivity Analysis
eFigure 1. The Construct-Diagram of the Stacking Model
eFigure 2. Area Under the Receiver Operating Characteristic Curve (AUROC), Sensitivity, and Specificity of Prediction Models for Predicting Cervical Intraepithelial Neoplasia Grade 2 or Worse in the Validation Dataset
eFigure 3. Calibration Plots of the Prediction Models Incorporating Epidemiological Factors, Pelvic Examination Results, and Human Papillomavirus (HPV) Genotypes for Predicting Cervical Intraepithelial Neoplasia Grade 3 or Worse (CIN3+) and CIN2+ Among Women Positive for High-Risk HPV Infection
eAppendix. R Code and User Guide

This supplementary material has been provided by the authors to give readers additional information about their work.

eTable 1. Candidate Predictors and Screening Outcomes in the Training and Validation Dataset

Candidate predictors / screening outcomes		Training data set, No. (%) (*n*=14553)	Validation data set, No. (%) (*n*=7167)
**Age (median, IQR), y**	-	51 (44-56)	49 (43-55)
**Gravidity (median, IQR)**	-	2 (2-3)	2 (2-3)
**Parity (median, IQR)**	-	2 (1-2)	2 (1-2)
**BMI[a, b]**	Normal	10360 (72.2)	5130 (71.7)
	Underweight	565 (3.9)	280 (3.9)
	Overweight	3423 (23.9)	1741 (24.3)
**Education**	Primary	7600 (52.2)	3076 (42.9)
	Middle	4081 (28.0)	1886 (26.3)
	High	1727 (11.9)	1185 (16.5)
	Graduate	1145 (7.9)	1020 (14.2)
**Insurance**	None	1022 (7.0)	303 (4.2)
	Employees	2175 (14.9)	2400 (33.5)
	Residents	11356 (78.0)	4464 (62.3)
**Family history of cancer**	No	14272 (98.1)	6997 (97.6)
	Yes	281 (1.9)	170 (2.4)
**Menopause**	No	7177 (49.3)	3915 (54.6)
	Yes	7376 (50.7)	3252 (45.4)
**Cervical screening[a]**	Never	11870 (81.6)	5033 (70.2)
	Within 3 years	2083 (14.3)	1677 (23.4)
	>3 years ago	592 (4.1)	455 (6.4)
**History of other cancers**	No	14133 (97.1)	6893 (96.2)
	Yes	420 (2.9)	274 (3.8)
**Post-coital bleeding**	No	14448 (99.3)	7063 (98.5)
	Yes	105 (0.7)	104 (1.5)
**Abnormal leukorrhea**	No	13836 (95.1)	6569 (91.7)
	Yes	717 (4.9)	598 (8.3)
**PE: vulva[a]**	Normal	14396 (98.9)	7045 (98.4)
	Abnormal	153 (1.1)	118 (1.6)
**PE: vagina[a]**	Normal	13806 (95.0)	6524 (91.1)
	Abnormal	733 (5.0)	639 (8.9)
**PE: secretion[a]**	Normal	11048 (75.9)	5604 (78.3)
	Abnormal	3499 (24.1)	1556 (21.7)
**PE: cervix[a]**	Normal	9135 (63.3)	3938 (55.2)
	Abnormal	5300 (36.7)	3200 (44.8)
**PE: uterus**	Normal	14142 (97.2)	6833 (95.3)
	Abnormal	411 (2.8)	334 (4.7)
**PE: adnexa**	Normal	14434 (99.2)	7112 (99.2)
	Abnormal	119 (0.8)	55 (0.8)
**Cervicitis**	No	13326 (91.6)	5952 (83.0)
	Yes	1227 (8.4)	1215 (17.0)
**Hysteromyoma**	No	14470 (99.4)	7080 (98.8)

© 2023 Xiao T et al. *JAMA Network Open*.

	Yes	83 (0.6)	87 (1.2)
**Vaginal status[a]**	I-II	9309 (67.0)	4697 (67.4)
	III-IV	4586 (33.0)	2277 (32.6)
**Trichomonas vaginalis[a]**	No	13559 (96.7)	6778 (97.0)
	Yes	464 (3.3)	208 (3.0)
**Candida [a]**	No	13420 (95.7)	6650 (95.2)
	Yes	603 (4.3)	336 (4.8)
**Gardnerella vaginalis[a]**	No	13965 (99.6)	6956 (99.6)
	Yes	58 (0.4)	30 (0.4)
**Clue cells[a]**	No	13827 (98.6)	6833 (97.8)
	Yes	196 (1.4)	153 (2.2)
**HPV genotypes**	HPV16	1724 (11.8)	830 (11.6)
	HPV16 + HPV18	43 (0.3)	12 (0.2)
	HPV16 + HPV18 + Other hrHPV genotypes	48 (0.3)	11 (0.2)
	HPV16 + Other hrHPV genotypes	761 (5.2)	392 (5.5)
	HPV18	509 (3.5)	224 (3.1)
	HPV18 + Other hrHPV genotypes	236 (1.6)	102 (1.4)
	Other hrHPV genotypes	11232 (77.2)	5596 (78.1)
**Primary outcome**	<CIN3	14204 (97.6)	7000 (97.7)
	CIN3+	349 (2.4)	167 (2.3)
**Secondary outcome**	<CIN2	13880 (95.4)	6939 (96.2)
	CIN2+	673 (4.6)	228 (3.8)

Abbreviations: BMI, body mass index; PE, pelvic examination; hrHPV, high-risk human papillomavirus;; <CIN3, including normal, CIN1, and CIN2; CIN3+, including CIN3 and cervical cancer; <CIN2, including normal and CIN1; CIN2+, including CIN2, CIN3, and cervical cancer.

[a] Predictors with missing values.

[b] BMI categories: Normal (18.5-25); Underweight (<18.5); Overweight (≥25).

© 2023 Xiao T et al. *JAMA Network Open*.

eTable 2. Predictors Selected by LASSO for CIN2+

Predictors	Participants in training data set, No. (%)				Participants in validation data set, No. (%)			
	Overall	<CIN2[a]	CIN2+[a]	*P* value	Overall	<CIN2[a]	CIN2+[a]	*P* value
No.	14553	13880	673	NA	7167	6939	228	NA
Age, median (IQR], y	51 (44, 56)	51 (44, 56)	49 (43, 55)	.008	49 (43, 55)	49 (43, 55)	48 (43, 54)	.19
Gravidity median (IQR)	2 (2, 3)	2 (2, 3)	3 (2, 4)	.04	2 (2, 3)	2 (2, 3)	3 (2, 3)	.97
BMI [b,c]								
Normal	10360 (72.2)	9876 (72.1)	484 (73.6)	.56	5,130 (71.7)	4968 (71.8)	162 (71.1)	.89
Underweight	565 (3.9)	537 (3.9)	28 (4.3)		280 (3.9)	272 (3.9)	8 (3.5)	
Overweight	3423 (23.9)	3277 (23.9)	146 (22.2)		1741 (24.3)	1683 (24.3)	58 (25.4)	
Insurance								
Not	1022 (7.0)	949 (6.8)	73 (10.8)	<.001	303 (4.2)	295 (4.3)	8 (3.5)	<.001
Employees	2175 (14.9)	2092 (15.1)	83 (12.3)		2400 (33.5)	2292 (33.0)	108 (47.4)	
Residents	11356 (78.0)	10839 (78.1)	517 (76.8)		4464 (62.3)	4352 (62.7)	112 (49.1)	
Family history of cancer								
No	14272 (98.1)	13622 (98.1)	650 (96.6)	.006	6997 (97.6)	6778 (97.7)	219 (96.1)	.17
Yes	281 (1.9)	258 (1.9)	23 (3.4)		170 (2.4)	161 (2.3)	9 (3.9)	
Menopause								
No	7177 (49.3)	6794 (48.9)	383 (56.9)	<.001	3915 (54.6)	3768 (54.3)	147 (64.5)	.003
Yes	7376 (50.7)	7086 (51.1)	290 (43.1)		3252 (45.4)	3171 (45.7)	81 (35.5)	
History of other cancers								
No	14133 (97.1)	13473 (97.1)	660 (98.1)	.16	6893 (96.2)	6674 (96.2)	219 (96.1)	>.99
Yes	420 (2.9)	407 (2.9)	13 (1.9)		274 (3.8)	265 (3.8)	9 (3.9)	
Cervical screening[b]								
Never	11870 (81.6)	11309 (81.5)	561 (83.5)	.44	5033 (70.2)	4873 (70.2)	160 (70.2)	.90
within 3 years	2083 (14.3)	1997 (14.4)	86 (12.8)		1677 (23.4)	1622 (23.4)	55 (24.1)	
>3 years ago	592 (4.1)	567 (4.1)	25 (3.7)		455 (6.4)	442 (6.4)	13 (5.7)	
PE: vulva[b]								
Normal	14396 (98.9)	13727 (98.9)	669 (99.4)	.32	7045 (98.4)	6823 (98.4)	222 (97.8)	.69
Abnormal	153 (1.1)	149 (1.1)	4 (0.6)		118 (1.6)	113 (1.6)	5 (2.2)	
PE: vagina[b]								
Normal	13806 (95.0)	13158 (94.9)	648 (96.4)	.09	6524 (91.1)	6311 (91.0)	213 (93.4)	.25
Abnormal	733 (5.0)	709 (5.1)	24 (3.6)		639 (8.9)	624 (9.0)	15 (6.6)	
PE: cervix[b]								
Normal	9135 (63.3)	8849 (64.3)	286 (42.8)	<.001	3938 (55.2)	3854 (55.8)	84 (36.8)	<.001
Abnormal	5300 (36.7)	4917 (35.7)	383 (57.2)		3200 (44.8)	3056 (44.2)	144 (63.2)	
PE: uterus								
Normal	14142 (97.2)	13486 (97.2)	656 (97.5)	.72	6833 (95.3)	6613 (95.3)	220 (96.5)	.50
Abnormal	411 (2.8)	394 (2.8)	17 (2.5)		334 (4.7)	326 (4.7)	8 (3.5)	
Cervicitis								
No	13326 (91.6)	12750 (91.9)	576 (85.6)	<.001	5952 (83.0)	5773 (83.2)	179 (78.5)	.08
Yes	1227 (8.4)	1130 (8.1)	97 (14.4)		1215 (17.0)	1166 (16.8)	49 (21.5)	

© 2023 Xiao T et al. *JAMA Network Open*.

Vaginal Status [b]								
I-II	9309 (67.0)	8929 (67.4)	380 (59.6)	<.001	4697 (67.4)	4567 (67.7)	130 (57.5)	.002
III-IV	4586 (33.0)	4328 (32.6)	258 (40.4)		2277 (32.6)	2181 (32.3)	96 (42.5)	
Gardnerella vaginalis [b]								
No	13965 (99.6)	13322 (99.6)	643 (99.8)	.47	6956 (99.6)	6731 (99.6)	225 (99.6)	>.99
Yes	58 (0.4)	57 (0.4)	1 (0.2)		30 (0.4)	29 (0.4)	1 (0.4)	
HPV genotypes								
16 only	1724 (11.8)	1373 (9.9)	351 (52.2)	<.001	830 (11.6)	708 (10.2)	122 (53.5)	<.001
16+18	43 (0.3)	37 (0.3)	6 (0.9)		12 (0.2)	9 (0.1)	3 (1.3)	
16+18+Other hrHPV genotypes	48 (0.3)	37 (0.3)	11 (1.6)		11 (0.2)	10 (0.1)	1 (0.4)	
16+Other hrHPV genotypes	761 (5.2)	644 (4.6)	117 (17.4)		392 (5.5)	346 (5.0)	46 (20.2)	
18 only	509 (3.5)	487 (3.5)	22 (3.3)		224 (3.1)	208 (3.0)	16 (7.0)	
18+Other hrHPV genotypes	236 (1.6)	228 (1.6)	8 (1.2)		102 (1.4)	100 (1.4)	2 (0.9)	
Other hrHPV genotypes	11232 (77.2)	11074 (79.8)	158 (23.5)		5596 (78.1)	5558 (80.1)	38 (16.7)	

Abbreviations: BMI, body mass index; PE, pelvic examination; hrHPV, high-risk human papillomavirus;.

[a] <CIN2, including normal and CIN1; CIN2+, including CIN2, CIN3, and carcinoma.

[b] Predictors with missing values.

[c] BMI categories: Normal (18.5-25); Underweight (<18.5); Overweight (≥25).

© 2023 Xiao T et al. *JAMA Network Open*.

eTable 3. Positive Likelihood Ratio and Negative Likelihood Ratio for All Models

Model	outcome = CIN3+		outcome = CIN2+	
	positive likelihood ratio	negative likelihood ratio	positive likelihood ratio	negative likelihood ratio
**LRM**				
Epidemiological factors + PE results	1.43	0.60	1.30	0.73
HPV genotypes only	4.96	0.26	4.85	0.29
All predictors	4.75	0.29	4.97	0.29
**RF**				
Epidemiological factors + PE results	1.55	0.52	1.44	0.83
HPV genotypes only	4.96	0.26	4.85	0.29
All predictors	4.60	0.27	3.31	0.23
**GBM**				
Epidemiological factors + PE results	1.61	0.68	1.28	0.76
HPV genotypes only	4.96	0.26	4.85	0.29
All predictors	4.96	0.26	4.42	0.26
**NB**				
Epidemiological factors + PE results	1.37	0.57	1.36	0.71
HPV genotypes only	4.96	0.26	4.85	0.29
All predictors	5.46	0.29	4.44	0.25
**NN**				
Epidemiological factors + PE results	1.51	0.57	1.32	0.69
HPV genotypes only	4.96	0.25	4.85	0.29
All predictors	3.42	0.20	4.57	0.26
**Stacking**				
Epidemiological factors + PE results	1.41	0.55	1.39	0.74
HPV genotypes only	4.96	0.26	4.85	0.29
All predictors	4.83	0.24	4.23	0.24

Abbreviations: LRM, logistic regression model; RF, random forest; GBM, gradient boosting machine; NB, naïve Bayes; NN, neural network.

eTable 4. Results of Sensitivity Analysis

Model[a]	CIN3+			CIN2+		
	AUROC (95% CI)	*Sen* (%)	*Spe* (%)	AUROC (95% CI)	*Sen* (%)	*Spe* (%)
LRM	0.82 (0.78-0.86)	73.2	83.0	0.78 (0.75-0.81)	64.3	84.8
RF	0.83 (0.80-0.87)	72.6	83.1	0.77 (0.74-0.80)	70.3	74.2
GBM	0.84 (0.81-0.87)	73.2	84.0	0.78 (0.75-0.81)	67.3	82.1
NB	0.84 (0.81-0.87)	69.8	86.2	0.77 (0.74-0.80)	67.7	82.1
NN	0.84 (0.81-0.87)	80.4	75.2	0.78 (0.75-0.81)	66.9	82.8
Stacking	0.84 (0.81-0.87)	74.3	83.4	0.78 (0.75-0.81)	69.2	80.6

Abbreviations: AUROC, the area under the receiver operating characteristic curve; sen, sensitivity; spe, specificity; LRM, logistic regression model; RF, random forest; GBM, gradient boosting machine; NB, naïve Bayes; NN, neural network.

[a] The model was constructed with HPV genotypes, epidemiological factors, and pelvic examination as factors.

© 2023 Xiao T et al. *JAMA Network Open*.

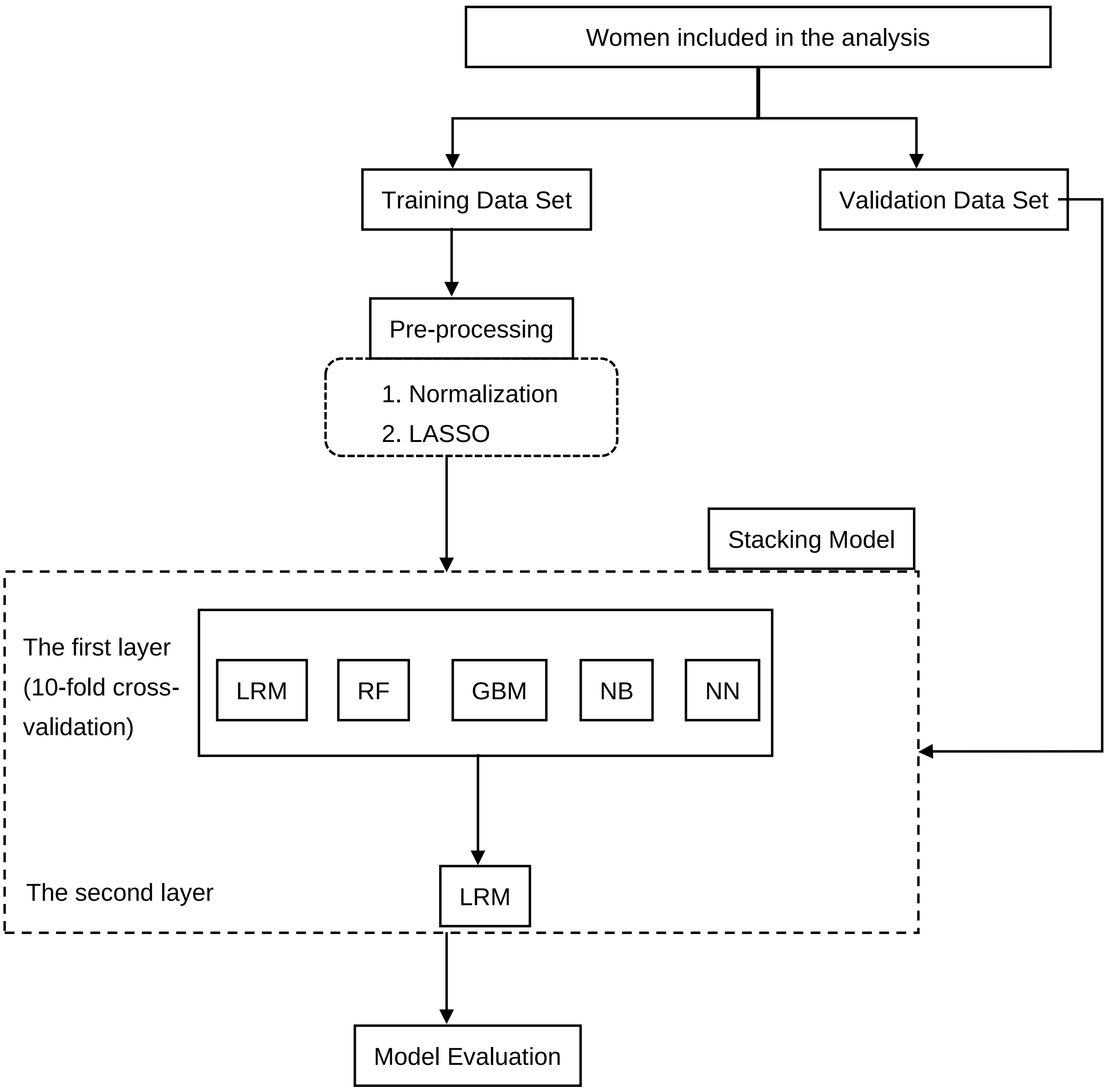

eFigure 1. The Construct-Diagram of the Stacking Model

LRM indicates logistic regression model; RF, random forest; GBM, gradient boosting machine; NB, naïve Bayes; and NN, neural network.

© 2023 Xiao T et al. *JAMA Network Open*.

Model	AUROC (95% CI)	sen	spe
**LRM**			
Epidemiological factors + PE results	0.59 (0.55−0.63)	62.2	52.1
HPV genotypes only	0.83 (0.80−0.86)	75.1	84.5
All predictors	0.84 (0.82−0.87)	75.1	84.9
**RF**			
Epidemiological factors + PE results	0.60 (0.56−0.63)	40.9	71.5
HPV genotypes only	0.83 (0.80−0.86)	75.1	84.5
All predictors	0.84 (0.81−0.87)	83.1	74.9
**GBM**			
Epidemiological factors + PE results	0.59 (0.55−0.63)	58.2	54.7
HPV genotypes only	0.83 (0.80−0.85)	75.1	84.5
All predictors	0.85 (0.82−0.87)	78.7	82.2
**NB**			
Epidemiological factors + PE results	0.59 (0.56−0.63)	61.3	54.8
HPV genotypes only	0.83 (0.80−0.86)	75.1	84.5
All predictors	0.84 (0.81−0.87)	79.1	82.2
**NN**			
Epidemiological factors + PE results	0.61 (0.57−0.64)	64.9	50.9
HPV genotypes only	0.83 (0.80−0.86)	75.1	84.5
All predictors	0.85 (0.82−0.88)	78.2	82.9
**Stacking**			
Epidemiological factors + PE results	0.60 (0.57−0.64)	55.6	59.9
HPV genotypes only	0.83 (0.80−0.86)	75.1	84.5
All predictors	0.85 (0.82−0.88)	80.4	81.0

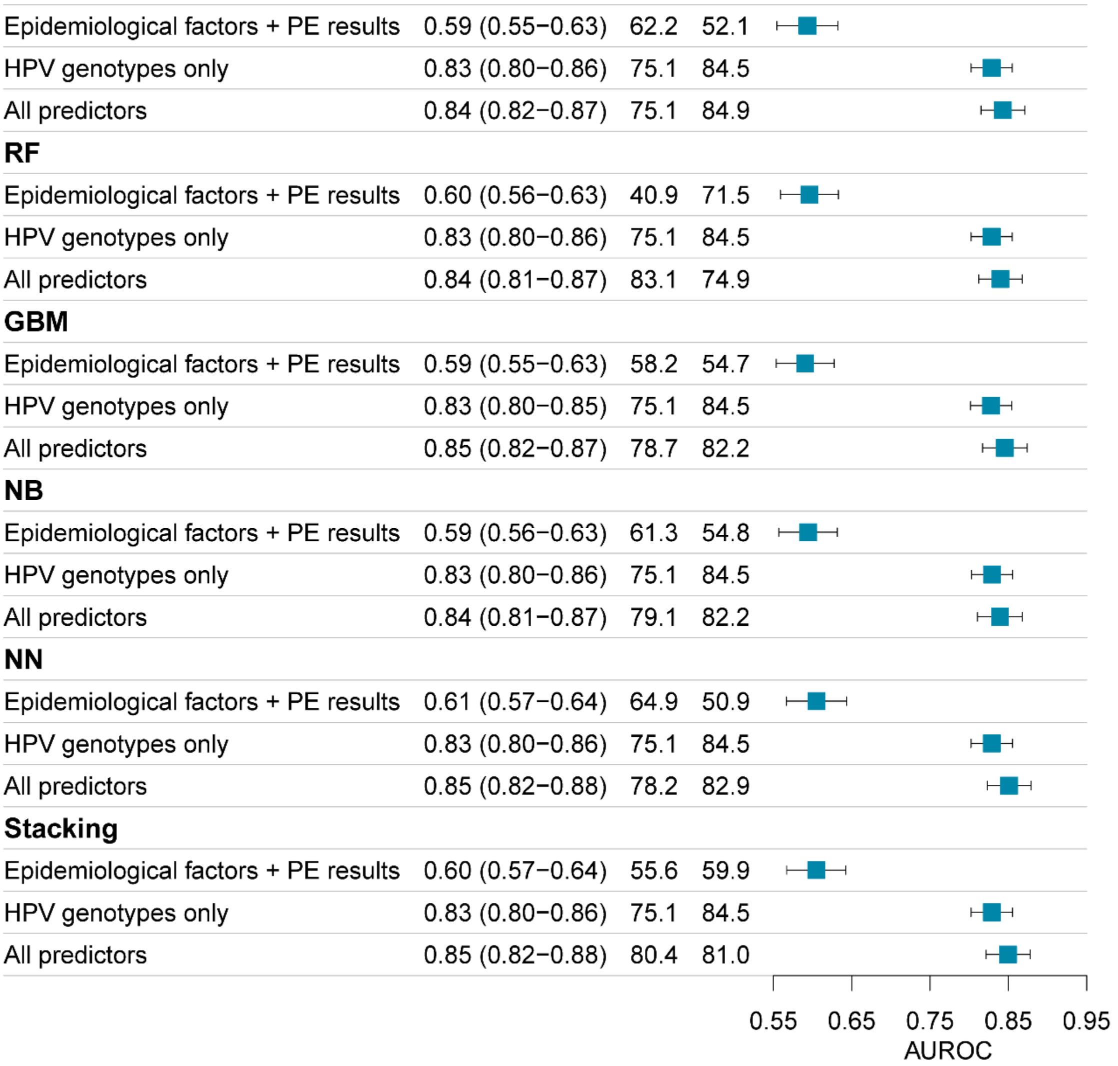

eFigure 2. Area Under the Receiver Operating Characteristic Curve (AUROC), Sensitivity, and Specificity of Prediction Models for Predicting Cervical Intraepithelial Neoplasia Grade 2 or Worse in the Validation Dataset

HPV indicates human papillomavirus; PE, pelvic examination; sen, sensitivity; spe, specificity; LRM, logistic regression model; RF, random forest; GBM, gradient boosting machine; NB, naïve Bayes; and NN, neural network.

© 2023 Xiao T et al. *JAMA Network Open.*

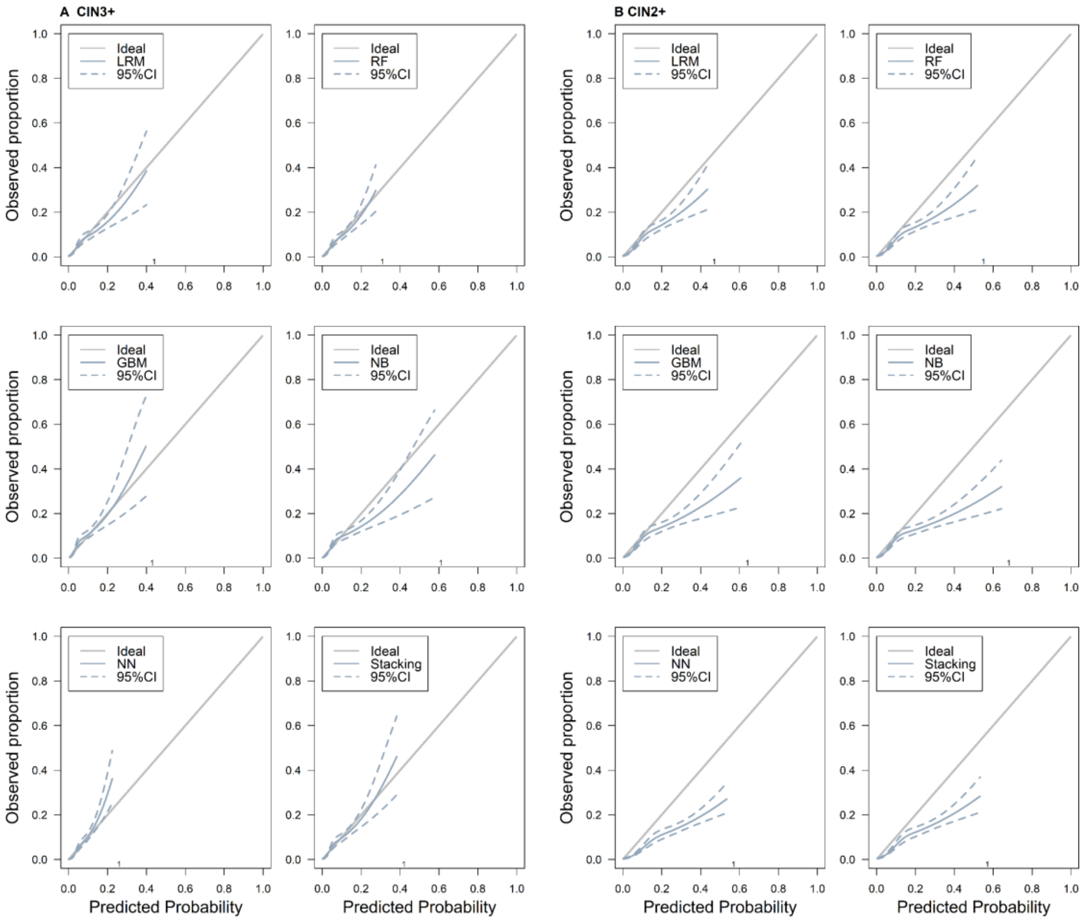

eFigure 3. Calibration Plots of the Prediction Models Incorporating Epidemiological Factors, Pelvic Examination Results, and Human Papillomavirus (HPV) Genotypes for Predicting Cervical Intraepithelial Neoplasia Grade 3 or Worse (CIN3+) and CIN2+ Among Women Positive for High-Risk HPV Infection

LRM indicates logistic regression model; RF, random forest; GBM, gradient boosting machine; NB, naïve Bayes; and NN, neural network.

eAppendix. R Code and User Guide

The R code is written in the form of a function which would easy for users to change or add predictors available in different settings. Simply by entering the training dataset, validation dataset, predictors, and outcome into the R function, the corresponding Area under the Receiver Operating Characteristic Curve (AUC), sensitivity, and specificity would be produced. Additionally, the final model (Stacking machine learning model) has been saved. The user can load this model and use it directly in future use, without retraining.

© 2023 Xiao T et al. *JAMA Network Open*.

Code:

```
library(caret) #To calculate the Confusion Matrix

library(pROC) #To calculate the AUC

library(h2o) #To construct the Stacking Model

library(tidyverse) # %>%


# Attempts to start and/or connect to an H2O instance
h2o.init(max_mem_size="12G")
# Define an R function
cervical_prediction<-function(train_data, test_data, predictors, outcome, seed=-1){
  h2o_train<-as.h2o(train_data)
  h2o_test<-as.h2o(test_data)
  n_features<-length(predictors)
# To build a logistic regression model
glm <- h2o.glm(
    x = predictors, y = outcome, training_frame = h2o_train, seed = seed,
    family=c("binomial"),link = "logit",
    remove_collinear_columns = TRUE, nfolds = 10, fold_assignment = "Modulo",
    keep_cross_validation_predictions = TRUE, lambda = 0,
    compute_p_values = TRUE,
    standardize = TRUE
  )
# To determine the threshold based on the maximum Youden index criterion
youden_glm<-h2o.predict(glm,newdata=h2o_train) %>% as.data.frame()
names(youden_glm)<-c("predict",table(youden_glm$predict)[1]%>%
    names(),table(youden_glm$predict)[2]%>% names(),"std")
auc_glm<-roc(train_data$outcome~youden_glm[,3], ci=TRUE, ci.alpha=0.95)
threshold_glm<-pROC::coords(auc_glm,best.method="youden","best")[1] %>% as.numeric()
```

# To predict the prediction outcome in the validation dataset

```
pre_glm<-h2o.predict(glm,newdata=h2o_test) %>% as.data.frame()
names(pre_glm)<-c("predict",table(youden_glm$predict)[1]%>%
  names(),table(youden_glm$predict)[2]%>% names(),"std")
pre_glm_class<-NA
pre_glm_class[pre_glm[,3]>threshold_glm]<-table(youden_glm$predict)[2]%>% names()
pre_glm_class[pre_glm[,3]<threshold_glm]<-table(youden_glm$predict)[1]%>% names()
```

# To calculate Sensitivity, Specificity, and AUC

```
sen_glm<-confusionMatrix(table(pre_glm_class,test_data$outcome),positive =
table(youden_glm$predict)[2]%>% names())$byClass[1] %>% as.numeric() %>%round(3) *100
spe_glm<-confusionMatrix(table(pre_glm_class,test_data$outcome),positive =
table(youden_glm$predict)[2]%>% names())$byClass[2] %>% as.numeric() %>%round(3) *100
auc_glm<-roc(test_data$outcome~pre_glm[,3], ci=TRUE, ci.alpha=0.95)
auc_glm<-paste0(round(auc_glm$auc,2)," (", round(auc_glm$ci,2)[1], "-", round(auc_glm$ci,2)[3],")")
```

# To build a random forest model

# Use the grid search to determine the model hyperparameters, or you can modify this part of the code to use the default hyperparameters directly. Learn more by ?h2o.randomForest().

```
 rf_grid <- list(            #To refine hyperparameter grid
   mtries = floor(n_features * c(.10, .15, .2, .25, .333, .4, .45)),
   min_rows = c(1, 3, 5, 10),
   max_depth = c(10, 20, 30),
   sample_rate = c(.55, .632, .70, .80)
 )

rf_search_criteria <- list(
   strategy = "RandomDiscrete",
```

```
    stopping_metric = "auc",
    stopping_tolerance = 0.001,   # Stop if improvement is < 0.1%
    stopping_rounds = 10,         # Over the last 10 models
    max_runtime_secs = 60*30      # or Stop search after 30 min.
  )

rf_grid <- h2o.grid(
    algorithm = "randomForest",
    grid_id = "rf_random_grid",
    x = predictors,
    y = outcome,
    training_frame = h2o_train,
    hyper_params = rf_grid,
    ntrees = 50,
    nfolds=10,
    search_criteria = rf_search_criteria,
    seed=seed
  )
# collect the results and sort by our model performance metric of choice (AUC)
rf_grid_perf <- h2o.getGrid(
    grid_id = "rf_random_grid",
    sort_by = "auc",
    decreasing = FALSE
  )
para_rf<-
    rf_grid_perf@summary_table[rf_grid_perf@summary_table$auc==max(rf_grid_perf@summary_ta
    ble$auc), ]
```

Random forest model

```
rf <- h2o.randomForest(
    x = predictors, y = outcome, training_frame = h2o_train,seed = seed,nfolds = 10,
    fold_assignment = "Modulo",
    keep_cross_validation_predictions = TRUE,
    keep_cross_validation_fold_assignment=TRUE,
    ntrees = 50, mtries = para_rf$mtries,
    max_depth = para_rf$max_depth,
    min_rows = para_rf$min_rows,
    sample_rate = para_rf$sample_rate,
    score_each_iteration = TRUE,
    stopping_metric = "mean_per_class_error",stopping_rounds = 50,
    stopping_tolerance = 0
  )

youden_rf<-h2o.predict(rf,newdata=h2o_train) %>% as.data.frame()
names(youden_rf)<-c("predict",table(youden_rf$predict)[1]%>%
    names(),table(youden_rf$predict)[2]%>% names())
auc_rf<-roc(train_data$outcome~youden_rf[,3], ci=TRUE, ci.alpha=0.95)
threshold_rf<-pROC::coords(auc_rf,best.method="youden","best")[1] %>% as.numeric()

pre_rf<-h2o.predict(rf,newdata=h2o_test) %>% as.data.frame()
names(pre_rf)<-c("predict",table(youden_rf$predict)[1]%>%
    names(),table(youden_rf$predict)[2]%>% names())
pre_rf_class<-NA
pre_rf_class[pre_rf[,3]>threshold_rf]<-table(youden_rf$predict)[2]%>% names()
pre_rf_class[pre_rf[,3]<threshold_rf]<-table(youden_rf$predict)[1]%>% names()
```

© 2023 Xiao T et al. *JAMA Network Open*.

```
sen_rf<-confusionMatrix(table(pre_rf_class,test_data$outcome),positive =
table(youden_rf$predict)[2]%>% names())$byClass[1] %>% as.numeric() %>%round(3) *100
spe_rf<-confusionMatrix(table(pre_rf_class,test_data$outcome),positive =
table(youden_rf$predict)[2]%>% names())$byClass[2] %>% as.numeric() %>%round(3) *100
auc_rf<-roc(test_data$outcome~pre_rf[,3], ci=TRUE, ci.alpha=0.95)
auc_rf<-paste0(round(auc_rf$auc,2)," (", round(auc_rf$ci,2)[1], "-", round(auc_rf$ci,2)[3],")")

# To build a gradient boosting machine model
# Use the grid search to determine the model hyperparameters, or you can modify this part of the code to use the default hyperparameters directly. Learn more by ?h2o.gbm().
gbm_grid <- list(
  sample_rate = c(0.5, 0.75, 1),
  learn_rate = c(0.01,0.03,0.05,0.07,0.1),
  min_rows = c(1,3,5,8,10),
  max_depth = c(3,5,7,10,30)
 )

gbm_search_criteria <- list(
  strategy = "RandomDiscrete",
  stopping_metric = "auc",
  stopping_tolerance = 0.001,
  stopping_rounds = 10,
  max_runtime_secs = 60*60
 )

gbm_grid <- h2o.grid(
  algorithm = "gbm",
  grid_id = "gbm_grid",
```

© 2023 Xiao T et al. *JAMA Network Open*.

```
    x = predictors,
    y = outcome,
    training_frame = h2o_train,
    hyper_params = gbm_grid,
    ntrees = 50,
    nfolds = 10,
    search_criteria = gbm_search_criteria,
    seed = seed
  )

gbm_grid_perf <- h2o.getGrid(
    grid_id = "gbm_grid",
    sort_by = "auc",
    decreasing = FALSE
  )

para_gbm<-
    gbm_grid_perf@summary_table[gbm_grid_perf@summary_table$auc==max(gbm_grid_perf@sum
    mary_table$auc), ]

gbm <-  h2o.gbm(
    x = predictors, y = outcome, training_frame = h2o_train, seed = seed, distribution="bernoulli",
    ntrees = 50, nfolds = 10, learn_rate = para_gbm$learn_rate, max_depth = para_gbm$max_depth,
min_rows = para_gbm$min_rows, sample_rate = para_gbm$sample_rate,
    fold_assignment = "Modulo",
    keep_cross_validation_predictions = TRUE,
    keep_cross_validation_fold_assignment=TRUE
  )
```

© 2023 Xiao T et al. *JAMA Network Open*.

```
  youden_gbm<-h2o.predict(gbm,newdata=h2o_train) %>% as.data.frame()
names(youden_gbm)<-c("predict",table(youden_gbm$predict)[1]%>%
   names(),table(youden_gbm$predict)[2]%>% names())
auc_gbm<-roc(train_data$outcome~youden_gbm[,3], ci=TRUE, ci.alpha=0.95)
threshold_gbm<-pROC::coords(auc_gbm,best.method="youden","best")[1] %>% as.numeric()

pre_gbm<-h2o.predict(gbm,newdata=h2o_test) %>% as.data.frame()
names(pre_gbm)<-c("predict",table(youden_gbm$predict)[1]%>%
   names(),table(youden_gbm$predict)[2]%>% names())
pre_gbm_class<-NA
pre_gbm_class[pre_gbm[,3]>threshold_gbm]<-table(youden_gbm$predict)[2]%>% names()
pre_gbm_class[pre_gbm[,3]<threshold_gbm]<-table(youden_gbm$predict)[1]%>% names()

sen_gbm<-confusionMatrix(table(pre_gbm_class,test_data$outcome),positive =
table(youden_gbm$predict)[2]%>% names())$byClass[1] %>% as.numeric() %>%round(3) *100
spe_gbm<-confusionMatrix(table(pre_gbm_class,test_data$outcome),positive =
table(youden_gbm$predict)[2]%>% names())$byClass[2] %>% as.numeric() %>%round(3) *100
auc_gbm<-roc(test_data$outcome~pre_gbm[,3], ci=TRUE, ci.alpha=0.95)
auc_gbm<-paste0(round(auc_gbm$auc,2)," (", round(auc_gbm$ci,2)[1], "-",
round(auc_gbm$ci,2)[3],")")
```

#To build naïve bayes model

```
nb <- h2o.naiveBayes(
   x = predictors, y = outcome, training_frame = h2o_train,
   laplace = 1,
   nfolds = 10,
   fold_assignment = "Modulo", keep_cross_validation_predictions = TRUE,
```

© 2023 Xiao T et al. *JAMA Network Open*.

```
    seed = seed)

youden_nb<-h2o.predict(nb,newdata=h2o_train) %>% as.data.frame()
names(youden_nb)<-c("predict",table(youden_nb$predict)[1]%>%
names(),table(youden_nb$predict)[2]%>% names())
auc_nb<-roc(train_data$outcome~youden_nb[,3], ci=TRUE, ci.alpha=0.95)
threshold_nb<-pROC::coords(auc_nb,best.method="youden","best")[1] %>% as.numeric()

pre_nb<-h2o.predict(nb,newdata=h2o_test) %>% as.data.frame()
names(pre_nb)<-c("predict",table(youden_nb$predict)[1]%>%
names(),table(youden_nb$predict)[2]%>% names())
pre_nb_class<-NA
pre_nb_class[pre_nb[,3]>threshold_nb]<-table(youden_nb$predict)[2]%>% names()
pre_nb_class[pre_nb[,3]<threshold_nb]<-table(youden_nb$predict)[1]%>% names()

sen_nb<-confusionMatrix(table(pre_nb_class,test_data$outcome),positive =
table(youden_nb$predict)[2]%>% names())$byClass[1] %>% as.numeric() %>%round(3) *100
spe_nb<-confusionMatrix(table(pre_nb_class,test_data$outcome),positive =
table(youden_nb$predict)[2]%>% names())$byClass[2] %>% as.numeric() %>%round(3) *100
auc_nb<-roc(test_data$outcome~pre_nb[,3], ci=TRUE, ci.alpha=0.95)
auc_nb<-paste0(round(auc_nb$auc,2)," (", round(auc_nb$ci,2)[1], "-", round(auc_nb$ci,2)[3],")")

# To build a neural network model
nn <- h2o.deeplearning(
    model_id="deeplearning",
    x = predictors,
    y = outcome, training_frame = h2o_train,distribution="bernoulli",
    seed =seed, reproducible = TRUE,
```

© 2023 Xiao T et al. *JAMA Network Open*.

```
    hidden=c(256,128,128),
    input_dropout_ratio=0.1,
    nfolds = 10,keep_cross_validation_fold_assignment = TRUE,
    fold_assignment = "Modulo", keep_cross_validation_predictions = TRUE
  )

youden_nn<-h2o.predict(nn,newdata=h2o_train) %>% as.data.frame()
names(youden_nn)<-c("predict",table(youden_nn$predict)[1]%>%
    names(),table(youden_nn$predict)[2]%>% names())
auc_nn<-roc(train_data$outcome~youden_nn[,3], ci=TRUE, ci.alpha=0.95)
threshold_nn<-pROC::coords(auc_nn,best.method="youden","best")[1] %>% as.numeric()

pre_nn<-h2o.predict(nn,newdata=h2o_test) %>% as.data.frame()
names(pre_nn)<-c("predict",table(youden_nn$predict)[1]%>%
    names(),table(youden_nn$predict)[2]%>% names())
pre_nn_class<-NA
pre_nn_class[pre_nn[,3]>threshold_nn]<-table(youden_nn$predict)[2]%>% names()
pre_nn_class[pre_nn[,3]<threshold_nn]<-table(youden_nn$predict)[1]%>% names()

sen_nn<-confusionMatrix(table(pre_nn_class,test_data$outcome),positive =
table(youden_nn$predict)[2]%>% names())$byClass[1] %>% as.numeric() %>%round(3) *100
spe_nn<-confusionMatrix(table(pre_nn_class,test_data$outcome),positive =
table(youden_nn$predict)[2]%>% names())$byClass[2] %>% as.numeric() %>%round(3) *100
auc_nn<-roc(test_data$outcome~pre_nn[,3], ci=TRUE, ci.alpha=0.95)
auc_nn<-paste0(round(auc_nn$auc,2)," (", round(auc_nn$ci,2)[1], "-", round(auc_nn$ci,2)[3],")")

# To build a Stacking model
eglm <- h2o.stackedEnsemble(
```

© 2023 Xiao T et al. *JAMA Network Open*.

```
    x = predictors, y = outcome, training_frame = h2o_train,
    metalearner_transform= "Logit",
    base_models = list(glm,gbm,rf,nb,nn),
    metalearner_algorithm = "glm",seed=seed
  )
youden_eglm<-h2o.predict(eglm,newdata=h2o_train) %>% as.data.frame()
names(youden_eglm)<-c("predict",table(youden_eglm$predict)[1]%>%
    names(),table(youden_eglm$predict)[2]%>% names())
auc_eglm<-roc(train_data$outcome~youden_eglm[,3], ci=TRUE, ci.alpha=0.95)
threshold_eglm<-pROC::coords(auc_eglm,best.method="youden","best")[1] %>% as.numeric()

pre_eglm<-h2o.predict(eglm,newdata=h2o_test) %>% as.data.frame()
names(pre_eglm)<-c("predict",table(youden_eglm$predict)[1]%>%
    names(),table(youden_eglm$predict)[2]%>% names())
pre_eglm_class<-NA
pre_eglm_class[pre_eglm[,3]>threshold_eglm]<-table(youden_eglm$predict)[2]%>% names()
pre_eglm_class[pre_eglm[,3]<threshold_eglm]<-table(youden_eglm$predict)[1]%>% names()

sen_eglm<-confusionMatrix(table(pre_eglm_class,test_data$outcome),positive =
    table(youden_eglm$predict)[2]%>% names())$byClass[1] %>% as.numeric() %>%round(3) *100
spe_eglm<-confusionMatrix(table(pre_eglm_class,test_data$outcome),positive =
    table(youden_eglm$predict)[2]%>% names())$byClass[2] %>% as.numeric() %>%round(3) *100
auc_eglm<-roc(test_data$outcome~pre_eglm[,3], ci=TRUE, ci.alpha=0.95)
auc_eglm<-paste0(round(auc_eglm$auc,2)," (", round(auc_eglm$ci,2)[1], "-",
round(auc_eglm$ci,2)[3],")")

# To save the Stacking model
model_path<-h2o.saveModel(object = eglm, path = getwd(), force = TRUE)
```

© 2023 Xiao T et al. *JAMA Network Open*.

```
print(model_path)

# To save the model result
result<-matrix(data =
    c("GLM",auc_glm,sen_glm,spe_glm,"RF",auc_rf,sen_rf,spe_rf,"GBM",auc_gbm,sen_gbm,spe_gbm
    ,"NB",auc_nb,sen_nb,spe_nb,"NN",auc_nn,sen_nn,spe_nn,"Stacking",auc_eglm,sen_eglm,spe_eglm
    ), nrow = 6, ncol=4, byrow = TRUE) %>% as.data.frame()
names(result)<-c("Model","AUC (95% CI)","Sensitivity","Specificity")
return(result, model_path)
}
# Entering the training dataset, validation dataset, predictors, and outcome into the R function
cervical_prediction, the corresponding AUC, sensitivity, and specificity would be produced.
#train_data: the training dataset, the outcome variable in the dataset must be named "outcome".
#test_data: the validation dataset, the outcome variable in the dataset must be named "outcome".
#predictors: predictors.
#outcome: predictive outcome.
#seed: Seed for random numbers. Defaults to -1 (time-based random number).

# Shut down the specified instance
h2o.shutdown()

# To load the Stacking model
model<- h2o.loadModel(model_path) # model_path is output by the R function cervical_prediction
```

© 2023 Xiao T et al. *JAMA Network Open*.
