# Supplementary material for: Use of Virus Genotypes in Machine Learning Diagnostic Prediction Models for Cervical Cancer in Women With High-Risk Human Papillomavirus Infection
Source: JAMA Netw Open. 2023 Aug 2;6(8):e2326890. doi: 10.1001/jamanetworkopen.2023.26890 (PMC10398410; doi:10.1001/jamanetworkopen.2023.26890)
Supplement: Supplement 2. — Data Sharing Statement [file jamanetwopen-e2326890-s002.pdf]

Data Sharing Statement

Xiao. Use of Virus Genotypes in Machine Learning Diagnostic Prediction Models for Cervical Cancer in Women With High-Risk Human Papillomavirus Infection. *JAMA Netw Open*. Published August 02, 2023. doi:10.1001/jamanetworkopen.2023.26890

Data
Data available: No

Additional Information
Explanation for why data not available: The individual participant-level data are not publicly available due to ethical and privacy concerns. The data used for the analysis are available upon email request from the corresponding authors (C.Q.O. and H.X.).
